# 3DFI: a pipeline to infer protein function using structural homology

**DOI:** 10.1093/bioadv/vbab030

**Published:** 2021-11-10

**Authors:** Alexander Thomas Julian, Anne Caroline Mascarenhas dos Santos, Jean-François Pombert

**Affiliations:** Department of Biology, Illinois Institute of Technology, Chicago, IL 60616, USA

## Abstract

**Summary:**

Inferring protein function is an integral part of genome annotation and analysis. This process is usually performed *in silico*, and most *in silico* inferences are based on sequence homology approaches, which can fail when in presence of divergent sequences. However, because protein structures and their biological roles are intertwined, protein function can also be inferred by searching for structural homology. Many excellent tools have been released in recent years with regards to protein structure prediction, structural homology searches and protein visualization. Unfortunately, these tools are disconnected from each other and often use a web server-based approach that is ill-suited to high-throughput genome-wide analyses. To help assist genome annotation, we built a structural homology-based pipeline called 3DFI (for tridimensional functional inference) leveraging some of the best structural homology tools. This pipeline was built with simplicity of use in mind and enables genome-wide structural homology inferences.

**Availability and implementation:**

3DFI is available on GitHub https://github.com/PombertLab/3DFI under the permissive MIT license. The pipeline is written in Perl and Python.

**Supplementary information:**

Supplementary data are available at *Bioinformatics Advances* online.

## 1 Introduction

To properly understand what an organism is truly capable of from a biological perspective, an in-depth knowledge of its genetic makeup and of the proteins it encodes is required. This knowledge can be acquired by genome sequencing projects, which can be divided into two phases: sequencing and annotation. While advances in high-throughput technologies have greatly sped up the sequencing phase, genome projects are often bottlenecked by the annotation phase. One of the challenges during annotation is to assign functions to predicted proteins. Because *in vitro* determination is too complex to be performed routinely, *in silico* methods are commonly used to infer protein functions, for example with BLAST-like programs, which search for similarity between protein queries and databases of known proteins.

However, sequence homology searches can fail to retrieve significant matches if the sequences being investigated are too divergent from those in the databases, which is often the case with understudied organisms or with newly emerging pathogens. While sensitivity can sometimes be increased by searching sequences against hidden Markov models with tools such as HHsearch (Steinegger *et al.*, 2019), these approaches are still reliant on a modicum of homology between queries and database sequences. Yet, because form often confers function in biology, two proteins with similar three-dimensional (3D) shapes yet very different amino acid compositions might still perform analogous biological functions, a property that can also be used to infer functions by looking for similar 3D patterns against databases of protein structures (e.g. RCSB PDB; Burley *et al.*, 2021).

While the idea of using structural homology to infer protein function has been around for a long time, it is now coming of age. Traditionally restricted to a select few projects due to the costs and efforts of solving protein structures experimentally, annotation efforts based on structural homology can now turn to computational predictions for help. Indeed, computational methods to predict the 3D structures of proteins have improved tremendously in accuracy, reliability and runtime since the first biennial Critical Assessment of Structure Prediction (CASP) competition in 1994 (Moult *et al.*, 1995), culminating in the impressive results by the AlphaFold2 team in CASP14, and both template- and deep learning-based predictors now produce structures that are often good enough to serve as queries for structural homology searches (e.g. with PDBeFOLD; Krissinel, 2007).

Unfortunately, several of these tools were designed around web interfaces that work well if users are interested in a handful of queries at a time but that are ill-suited to the scope of entire genome projects. To help automate genome-wide annotation efforts based on structural homology, we built a simple pipeline named 3DFI—for tridimensional functional inference—that can perform template- and deep-learning protein structure modelling with select predictors, run structural homology searches and align/visualize the corresponding matches. This pipeline is freely available on GitHub under the permissive MIT license.

## 2 Pipeline overview

The 3DFI pipeline uses three master scripts to install most of the dependencies, download (or create) the required databases and search for structural homologues in the 3D space (setup_3DFI.pl, create_3DFI_db.pl and run_3DFI.pl, respectively).

**Fig. 1.**
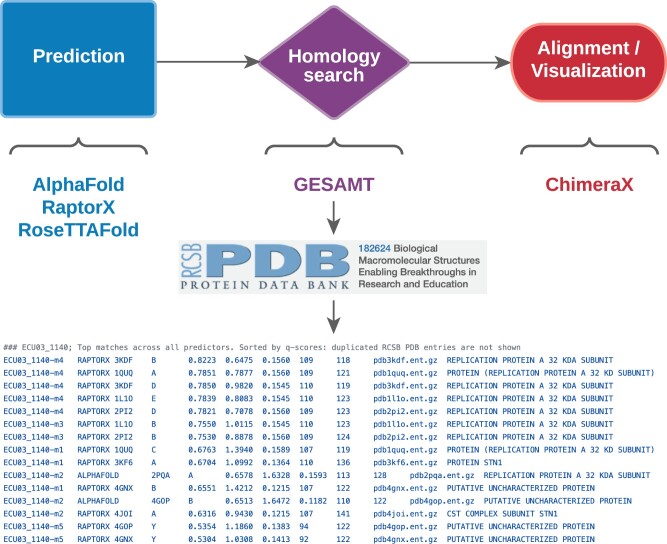
Overview of the 3DFI pipeline. The pipeline contains three main steps: protein structure prediction, structural homology searches and alignment between predicted protein structures and their putative structural homologues. Protein structures can be predicted with AlphaFold2, RaptorX and/or RoseTTAFold. Structural homology searches are performed with GESAMT against experimental structures from the RCSB PDB database with matches stored in tab-delimited files. Alignments are performed with ChimeraX

The search for structural homologues with 3DFI can be divided into three steps: (i) protein structure prediction; (ii) structural homology searches; and (iii) alignment/visualization of the resulting matches (Fig. 1). All three steps can be performed with run_3DFI.pl from a single command line. Visualization of the matches found with 3DFI can also be performed independently with run_visualizations.pl.

The 3DFI pipeline currently supports a total of three distinct protein structure predictors: the template-based RaptorX (Ma *et al.*, 2013) and the deep-learning-based AlphaFold2 (Jumper *et al.*, 2021) and RoseTTAFold (Baek *et al.*, 2021). Predicted protein structures are queried against experimentally resolved structures from the RCSB PDB database (Burley *et al.*, 2021) with the General Efficient Structural Alignment of Macromolecular Targets (GESAMT) algorithm (Krissinel, 2012) from the CCP4 package (https://www.ccp4.ac.uk/). Alignments between protein queries and their putative structural homologues are performed with ChimeraX (Pettersen *et al.*, 2021), which is also used to visualize the alignments.

## 3 Recommended hardware

To run 3DFI with deep-learning tools, a workstation equipped with a Compute Unified Device Architecture (CUDA)-enabled graphics processing unit (GPU) (24 GB+ VRAM), a fast multicore central processing unit (CPU), at least 64 GB of RAM and a fast 4 Tb+ solid-state drive (SSD) is recommended. AlphaFold2 (Jumper *et al.*, 2021) and RoseTTAFold (Baek *et al.*, 2021) leverage the NVIDIA CUDA framework to perform computations and, while both tools can be run without GPU acceleration, the process is much slower and therefore not recommended. Although small proteins might fit within 8 GB of VRAM (as in the case example below), we recommend at least 24 GB of VRAM for large proteins. Additionally, AlphaFold2 can use a substantial amount of system memory even with its –reduced_dbs preset, and thus a minimum of 64 GB of RAM is recommended. Because both AlphaFold2 and RoseTTAFold utilize the I/O intensive HHblits from HH-suite3 (Steinegger *et al.*, 2019), computation times can also be improved considerably by storing the databases on a fast SSD. In contrast, the template-based RaptorX (Ma *et al.*, 2013) is CPU bound, leverages PSI-BLAST searches and can be performed on machines with more modest specifications.

## 4 Software requirements

The 3DFI pipeline was tested on Linux workstations (Fedora 33/34). The pipeline is built in Perl with a few helper scripts in Python and was designed for modularity. The additional Perl module PerlIO::gzip is required to handle compressed files on the fly. Full containerization was not possible due to licensing restrictions of some of the dependencies. The 3DFI pipeline leverages aria2 (https://aria2.github.io/) and rsync (https://rsync.samba.org/) to facilitate downloads. Docker (https://www.docker.com/) is required for AlphaFold2 whereas Conda (https://docs.conda.io/) is required for RoseTTAFold. The latter also requires a PyRosetta 4 license (https://www.pyrosetta.org/). Users interested in RaptorX will need a Modeller license (https://salilab.org/modeller). The CCP4 package (https://www.ccp4.ac.uk/) is required for structural homology searches with GESAMT. ChimeraX (https://www.cgl.ucsf.edu/chimerax/) is required for structure alignments and visualization.

## 5 Case example—unknown proteins from microsporidia

*Encephalitozoon* species are fungal-like NIAID category B pathogens belonging to the phylum Microsporidia with small proteomes of about 2000 proteins (Pombert *et al.*, 2015). The proteins encoded in *Encephalitozoon* genomes are highly divergent, such that roughly half of their proteomes cannot be identified using sequence-based homology approaches. As a case example, three small proteins (ECU03_1140, ECU06_1350 and ECU08_1425) from *Encephalitozoon cuniculi* that cannot be identified by sequence homology are provided with 3DFI. These proteins were selected because: (i) running InterProScan searches on their sequences returned no results; (ii) they can be folded within 8 GB of VRAM; and (iii) they serve as good examples of the benefits and limitations of using structural homology approaches to infer protein function.

**Fig. 2.**
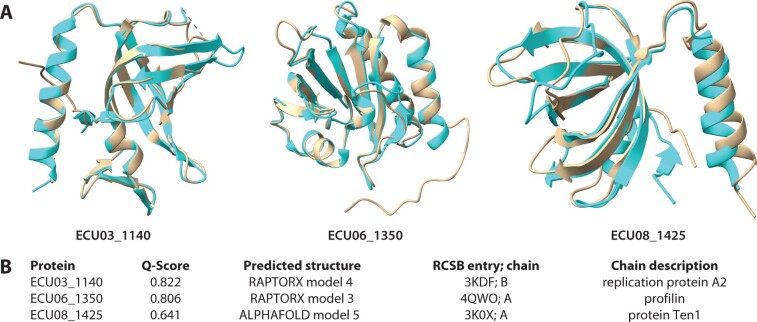
Example of structural homologues identified with 3DFI. (**a**) Alignments between protein structures predicted with RaptorX or AlphaFold2 (in cyan) and their top match from the RCSB PDB database (in gold). The top matches between the various models folded by all three predictors (AlphaFold2, RaptorX and RoseTTAFold) and their structural homologues were selected by decreasing Q-score values, as calculated with GESAMT. The proteins shown are provided as examples with the 3DFI pipeline. (**b**) Information corresponding to each of the alignments. This information is displayed in the interactive prompts during the 3DFI visualization step

Using 3DFI, we were able to predict the structures of these proteins, find structural homologues in the RCSB PDB database and align the predicted proteins to their structural homologues (Fig. 2, Supplementary Data S1). From these structural homologues, we were able to infer that ECU03_1140 is involved in DNA repair/maintenance (but see Section 6 for a succinct interpretation of this result), ECU06_1350 is involved in actin polymerization dynamics and ECU08_1425 is involved in the protection of telomeres.

On our AMD workstation (Ryzen 5950X; NVIDIA RTX A6000; 128 GB RAM; NVME SSD), the process from start to finish took 1 h and 45 min using all three protein structure predictors (AlphaFold2, RoseTTAFold and RaptorX). The same process took 4 h and 25 min on our Intel workstation (Xeon E5-2640; NVIDIA GTX 1070; 128 GB RAM; 7200 RPM hard drive). In contrast, running the pipeline on the same machines using AlphaFold2 as the only protein structure predictor took 51 min and 1 h 59 min, respectively. If a single predictor is to be used, we recommend AlphaFold2 based on its performance in the CASP14 competition (https://predictioncenter.org/casp14/); in cases where structures predicted with AlphaFold are suboptimal, users may want to try RaptorX (the template-based approach of RaptorX may work where deep-learning ones fail). Folding of the complete *E. cuniculi* proteome with AlphaFold2 averaged about 50 proteins per day on our NVIDIA RTX A6000-equipped workstation, and we anticipate that most users will want to use a single predictor for large datasets. We also anticipate that most users will want to run the visualization step independently of the computation steps. This can be done with run_visualizations.pl on lower-specced machines such as laptops.

## 6 Discussion

We initially implemented the 3DFI pipeline to help us annotate highly divergent proteins from obligate intracellular human parasites from the genus *Encephalitozoon*, a group of organisms for which half the proteome is unknown. Although the *Encephalitozoon* proteome is extremely small for a eukaryote with only 2000 or so proteins, even such a small number of queries made manual inferences an unsavoury proposition, and the 3DFI pipeline was built from a need for automation.

At the time, deep-learning folding methods were not yet available and the 3DFI pipeline was prototyped around the template-based RaptorX (Ma *et al.*, 2013), which showed excellent results in Continuous Automated Model Evaluation (CAMEO) assessments (https://www.cameo3d.org/). Since, we added support for the deep-learning predictors AlphaFold2 (Jumper *et al.*, 2021) and RoseTTAFold (Baek *et al.*, 2021) as they were released.

Our quest for automation led us to GESAMT (Krissinel, 2012) for structural homology searches, as the web-based interface of PDBeFOLD made it a non-starter. GESAMT was designed as an improvement on the Secondary Structure Matching (SSM) algorithm used in PDBeFOLD and returns similar (if not better) results when performing searches. In both tools, a Q-score value (from 0 to 1) is returned as a measure of similarity between 3D structures. We found in our tests that any Q-score value above 0.5 usually implies a good structural homology and that decent matches (albeit partial) can sometimes be found even with Q-scores near the 0.3 cut-off. This observation builds on our work with complete Microsporidia proteomes, but users may prefer different cut-offs for other organisms. At a 0.3 cut-off, we were able to find putative structural homologues for about 68% of the *E. cuniculi* proteins (results not shown). A clear advantage of using GESAMT instead of PDBeFOLD is that custom databases can be generated with GESAMT, whereas PDBeFOLD users are restricted to the options implemented on its website. A common strategy with sequence homology approaches is to use bidirectional searches to help ascertain matches, and this strategy is also possible here with GESAMT (a user could create a database of predicted proteins, then query known proteins against it for possible matches; see Supplementary Data S2 for an example of how to do this).

Our exploration of the structural matches and their significance further led us to the realization that adding a semi-automated visualization step to inspect the alignments between queries and putative matches would be very valuable for Q-scores that are on the fence. As such, we implemented an optional visualization step that leverages the excellent ChimeraX (Pettersen *et al.*, 2021) molecular visualization program for curation by expert users.

The idea of using structural homology to help with protein annotation is becoming increasingly attractive with the latest improvements to folding algorithms, and we expect that this approach will become an integral part of genome sequencing and annotation efforts. However, caution should be applied when using structural homology to infer protein function. In our examples, the best structural homologue found for ECU03_1140—based on Q-scores alone—is replication protein A2 (Rpa2) even though ECU03_1140 is most likely to be Stn1 for the following reasons: (i) Stn1 and Rpa2 are structural analogues and Rpa2 already has been identified by sequence homology approaches in the *Encephalitozoon* proteomes; (ii) Stn1 was found as a match to ECU03_1140 by 3DFI albeit with a lower Q-score (see Fig. 1; Supplementary Data S1); and (iii) Stn1 protects telomeres from degradation (i.e. DNA maintenance) with the help of Ten1, which we also discovered with 3DFI (Fig. 2; ECU08_1425). Thus, inferring protein function based solely on structural information could lead to erroneous conclusions, and this process should not be used in a vacuum but rather as a complement to other existing approaches.

What we aimed to achieve when building 3DFI was to simplify the process of inferring protein function by structural homology by leveraging automation combined with the best tools available for each task, and we believe that the 3DFI pipeline will be helpful to the genomic community.

### Author contributions

J.-F.P. designed the pipeline; J.-F.P., A.T.J. and A.C.M.d.S. wrote the software and tested the pipeline; J.-F.P., A.T.J. and A.C.M.d.S. wrote and reviewed the manuscript.

## Funding

This work was supported in part by a grant from the National Institute of Allergy and Infectious Diseases of the National Institutes of Health [grant number R15AI128627] to J.-F.P. The content is solely the responsibility of the authors and does not necessarily represent the official views of the National Institutes of Health.

*Conflict of Interest*: none declared.

## Supplementary Material

vbab030_Supplementary_Data
