# Supplementary material for: 3DFI: a pipeline to infer protein function using structural homology
Source: Bioinform Adv. 2021 Nov 10;1(1):vbab030. doi: 10.1093/bioadv/vbab030 (PMC9162058; doi:10.1093/bioadv/vbab030)
Supplement: vbab030_Supplementary_Data [file vbab030_supplementary_data.zip › Supplementary_Data_1_3DFI.pdf]

Supplementary Data 1: Step-by-step walkthrough of how to use the 3DFI pipeline with the provided case examples. This walkthrough was tested on Fedora 33/34 Linux installations with 3DFI version 0.8.6. This document contains the following sections: installation of the 3DFI pipeline, using the 3DFI pipeline, and interpreting the results of the 3DFI pipeline. Up-to-date instructions can be found on GitHub (https://github.com/PombertLab/3DFI).

Table of Contents

Installation of the 3DFI pipeline 3

Downloading the 3DFI pipeline with git and installing basic dependencies 3

Installing protein structure predictors with setup_3DFI.pl 3

Installing Modeller 4

Installing GESAMT 4

Installing ChimeraX 4

Installing databases with create_3DFI_db.pl 4

Using the 3DFI pipeline 5

Using 3DFI to predict protein structures, then find and align structural homologs 5

Using run_visualizations.pl to inspect alignments between predicted proteins and their homologs . 7

Interpreting the results of the 3DFI pipeline 11

Installation of the 3DFI pipeline

Downloading the 3DFI pipeline with git and installing basic dependencies

The 3DFI pipeline can be downloaded from GitHub as follows:

```
git clone https://github.com/PombertLab/3DFI.git
```

The 3DFI pipeline leverages aria2 to download data files and uses the PerlIO::gzip module to handle compressed .gzip files on the fly. To install these two dependencies on Fedora, use the following command:

```
sudo dnf install aria2 perl-PerlIO-gzip
```

Installing protein structure predictors with setup_3DFI.pl

Three protein structure predictors are supported by 3DFI: AlphaFold2, RaptorX, and RoseTTAFold. These can be installed automatically with setup_3DFI.pl. 3DFI utilizes a customized version of AlphaFold2 which should be installed with setup_3DFI.pl. A PyRosetta 4 license is required for RoseTTAFold. The license is necessary to download the PyRosetta python3.7 tarball (e.g. PyRosetta4.Release.python37.linux.release-297.tar.bz2), which can be acquired from https://www.pyrosetta.org/.

The setup_3DFI.pl script creates environment variables that point to important components used in the pipeline. A user-provided configuration file will store the environment variables. An empty configuration file can be provided (e.g. 3DFI.sh), or alternatively, users may provide an existing configuration file (e.g. ~/.bashrc).

The following command installs all three structure predictors with setup_3DFI.pl:

```
cd 3DFI/
./setup_3DFI.pl \
  -c ~/.bashrc \
  -d /media/databases/3DFI \
  -i alphafold raptorx rosettafold \
  -pyr ~/Downloads/PyRosetta4.Release.python37.*.tar.bz2
```

The configuration file is specified with the (-c) flag. The location of where databases will be stored is specified with (-d). If users plan to utilize RoseTTAFold, the location of the downloaded PyRosetta tarball must be specified with (-pyr).

An example of 3DFI environment variables stored in the user specified configuration file is as follows:

```
##################################################
# 3DFI environment variables

export TDFI_HOME=/media/FatCat_2/opt/3DFI
export TDFI_DB=/media/SSD4/3DFI

export RAPTORX_HOME=/media/FatCat_2/opt/3DFI/3D/RaptorX
export ROSETTAFOLD_HOME=/media/FatCat_2/opt/3DFI/3D/RoseTTAFold
export ALPHAFOLD_HOME=/media/FatCat_2/opt/3DFI/3D/alphafold
export PYTHONPATH=$PYTHONPATH:/media/FatCat_2/opt/3DFI/3D/alphafold/python/
```

Installing Modeller

RaptorX requires Modeller. Users can request a license for Modeller and download it from https://salilab.org/modeller/. The commands to install Modeller are as follows:

```
LICENSE=XXXXX                              ## replace XXXXX by Modeller license
MODELLER=modeller-10.1-1.x86_64.rpm
sudo env KEY_MODELLER=$LICENSE rpm -Uvh $MODELLER
```

Installing GESAMT

3DFI searches for homology in the 3D space with GESAMT from the CCP4 package. Users can download the CCP4 package from https://www.ccp4.ac.uk/. After installation, the CCP4 package location must be added to the path, e.g.:

```
export CCP4=/opt/xtal/CCP4/ccp4-7.1/bin/   ## replace with install location
export PATH=$PATH:$CCP4
```

Installing ChimeraX

ChimeraX is used for the alignment and visualization of predicted protein structures to their putative structural homologs. ChimeraX can be downloaded from https://www.rbvi.ucsf.edu/chimerax/ , and installed as follows:

```
sudo dnf install ucsf-chimerax-*.rpm
```

Installing databases with create_3DFI_db.pl

The required databases for protein prediction and homology searches can be downloaded with create_3DFI_db.pl. Because AlphaFold2 and RoseTTAFold uses the I/O intensive HHblits tool from the HH-suite3 package, a fast SSD is recommended to store the databases. The complete set of databases can be downloaded with the following command:

```
cd $TDFI_HOME
./create_3DFI_db.pl --all --delete
```

If the (--delete) flag is invoked as above, the downloaded archives are removed after the unpacking process. Note that downloading all databases as above will require at least 3.4Tb of disk space.

```
RSCB PDB                           # 39 Gb / 42 Gb inflated
BFD (AlphaFold/RoseTTAFold)        # 272 Gb / 1.8 Tb inflated
AlphaFold (minus BFD)              # 176 Gb / 0.6 Tb inflated
RoseTTAFold (minus BFD)            # 146 Gb / 849 Gb inflated
RaptorX                            # 37 Gb / 76 Gb inflated
```

Specific databases can also be downloaded independently by invoking the corresponding command line switches:

```
--rcsb        # RCSB PDB/GESAMT
--alpha       # AlphaFold2
--raptorx     # RaptorX
--rosetta     # RoseTTAFold
```

Using the 3DFI pipeline

The 3DFI pipeline was designed to assist in the protein annotation process. For the example proteins included with 3DFI, no putative function could be inferred by sequence-based homology approaches.

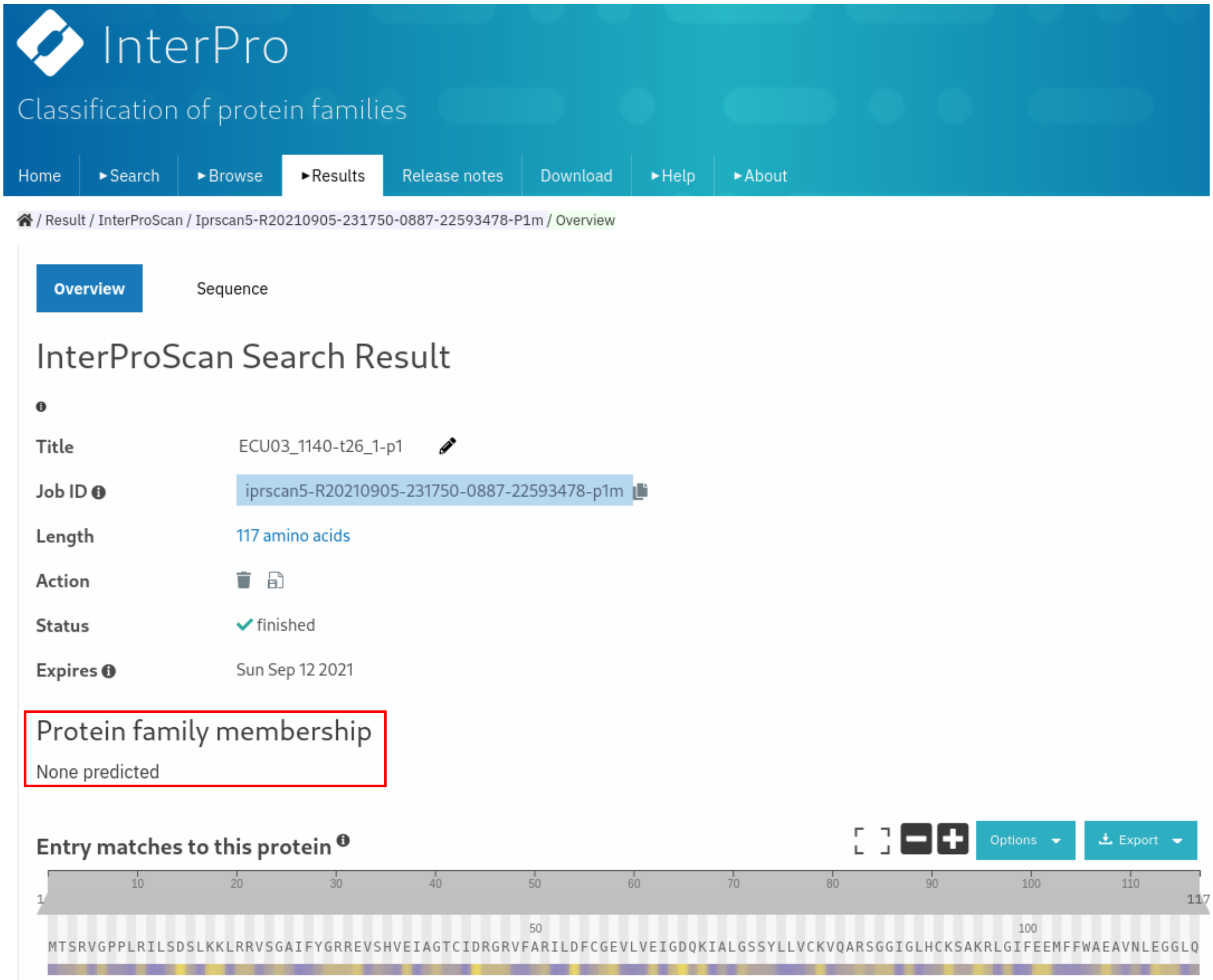

Fig. S1. Failed sequence-based functional inference. In this example, InterProScan was unable to assign putative functions (red rectangle) to example protein ECU03_1140.

Using 3DFI to predict protein structures, then find and align structural homologs

The run_3DFI.pl master script is comprised of three functional components. The first component predicts the 3D structures of the given proteins. AlphaFold2 and RoseTTAFold utilize a deep learning approach to structure prediction, whereas RaptorX utilizes a template-based approach. The next component leverages GESAMT to identify structural homologs to the predicted structures. The last component utilizes ChimeraX to align the predicted structures to their homologs found through GESAMT searches.

To use run_3DFI.pl with all predictors on the examples provided with 3DFI, use the following command:

```
export RESULTS=~/Results_3DFI            ## Change to desired output location

run_3DFI.pl \
  -f $TDFI_HOME/Examples/FASTA/*.fasta \
  -o $RESULTS \
  -c 16 \
  -p alphafold rosettafold raptorx
```

The run_3DFI.pl script takes fasta sequence files as inputs (-f flag) and places the results in the specified output folder (-o flag). For non-GPU dependent steps, the number of cores available for utilization can be specified with (-c). Protein structure predictors can be selected with the (-p) flag.

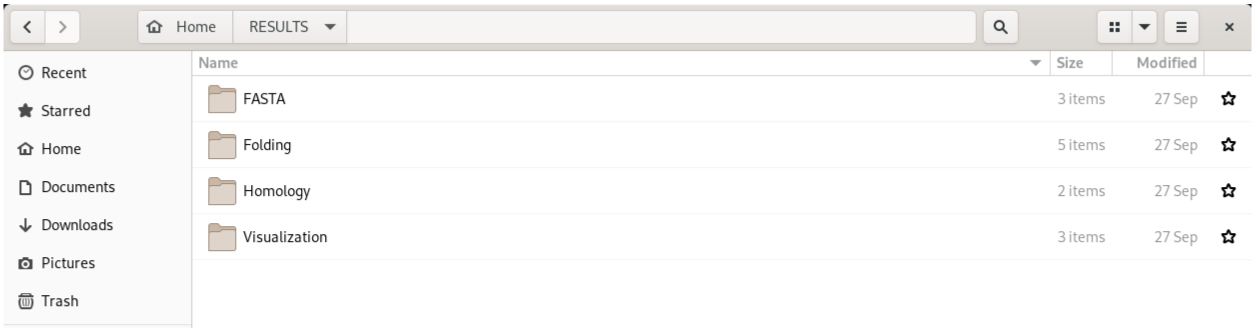

Fig. S2. Output of run_3DFI.pl. The output of run_3DFI.pl is split into four folders: 1) FASTA contains the per-protein fasta files; 2) Folding contains predicted 3D structures; 3) Homology contains the results of GESAMT structural homology searches on the predicted proteins; and 4) Visualization contains the alignments between the predicted proteins and their corresponding structural homolog(s).

Structural homologs identified by 3DFI are ranked by decreasing Q-score values. More than one model can be generated for each protein. Under Homology/GESAMT/, two files are created for each predictor: one ranked on a per-model basis, and the other ranked on a per-protein basis. Additionally, one master file (All_GESAMT_matches_per_protein.tsv) is created. This master file ranks all models created by all predictors and their structural homologs on a per-protein basis.

The content of All_GESAMT_matches_per_protein.tsv should look like the following (some columns are not shown for brevity).

```
ECU03_1140-m4 RAPTORX    3KDF   B   0.8223   … REPLICATION PROTEIN A 32 KDA SUBUNIT
ECU03_1140-m4 RAPTORX    1QUQ   A   0.7851   … PROTEIN (REPLICATION PROTEIN A 32 KD SUBUNIT)
ECU03_1140-m4 RAPTORX    3KDF   D   0.7850   … REPLICATION PROTEIN A 32 KDA SUBUNIT
ECU03_1140-m4 RAPTORX    1L1O   E   0.7839   … REPLICATION PROTEIN A 32 KDA SUBUNIT
ECU03_1140-m4 RAPTORX    2PI2   D   0.7821   … REPLICATION PROTEIN A 32 KDA SUBUNIT
ECU03_1140-m3 RAPTORX    1L1O   B   0.7550   … REPLICATION PROTEIN A 32 KDA SUBUNIT
ECU03_1140-m3 RAPTORX    2PI2   B   0.7530   … REPLICATION PROTEIN A 32 KDA SUBUNIT
ECU03_1140-m1 RAPTORX    1QUQ   C   0.6763   … PROTEIN (REPLICATION PROTEIN A 32 KD SUBUNIT)
ECU03_1140-m1 RAPTORX    3KF6   A   0.6704   … PROTEIN STN1
ECU03_1140-m2 ALPHAFOLD  2PQA   A   0.6578   … REPLICATION PROTEIN A 32 KDA SUBUNIT
ECU03_1140-m1 RAPTORX    4GNX   B   0.6551   … PUTATIVE UNCHARACTERIZED PROTEIN
ECU03_1140-m2 ALPHAFOLD  4GOP   B   0.6513   … PUTATIVE UNCHARACTERIZED PROTEIN
ECU03_1140-m2 RAPTORX    4JOI   A   0.6316   … CST COMPLEX SUBUNIT STN1
ECU03_1140-m5 RAPTORX    4GOP   Y   0.5354   … PUTATIVE UNCHARACTERIZED PROTEIN
ECU03_1140-m5 RAPTORX    4GNX   Y   0.5304   … PUTATIVE UNCHARACTERIZED PROTEIN
```

Using run_visualizations.pl to inspect alignments between predicted proteins and their homologs

The run_visualizations.pl script presents the contents of the All_GESAMT_matches_per_protein.tsv output file in an interactive and easily digestible manner. Additionally, users are able to view the various models generated by predictors for each protein and view alignments between predicted structures and their homologs.

Launching run_visualizations.pl

The run_visualizations.pl script can be launched immediately after the creation of alignments by specifying the (-v) flag when using run_3DFI.pl as follows:

```
run_3DFI.pl \
  -f $TDFI_HOME/Examples/FASTA/*.fasta \
  -o $RESULTS \
  -c 16 \
  -p alphafold rosettafold raptorx \
  -v
```

Users may also launch run_visualizations.pl standalone via the following command:

```
run_visualizations.pl -r $RESULTS
```

An example of the interactive prompt of run_visualizations.pl is provided below (some columns in the table are hidden for brevity).

```
### ECU03_1140 has 15 matches. ###

  - Currently in best match mode
  - Viewing only proteins with matches

|==================================================================================================|
  Selection   Q-Score     Predicted Structure    …    Structural Homolog Description
|==================================================================================================|
      1        0.822       RAPTORX => Model 4    …    REPLICATION PROTEIN A 32 KDA SUBUNIT
      2        0.785       RAPTORX => Model 4    …    PROTEIN (REPLICATION PROTEIN A 32 KD SUBUNIT)
      3        0.785       RAPTORX => Model 4    …    REPLICATION PROTEIN A 32 KDA SUBUNIT
      4        0.784       RAPTORX => Model 4    …    REPLICATION PROTEIN A 32 KDA SUBUNIT
      5        0.782       RAPTORX => Model 4    …    REPLICATION PROTEIN A 32 KDA SUBUNIT
|==================================================================================================|

Selectable Options:

  [1-5] Open corresponding match file
  [M] To select predicted structure

  [A] Show ALL matches
  [C] Include predicted structures without matches

  [N] Proceed to the next locus
  [P] Proceed to the previous locus
  [J] Jump to a selected locus

  [H] Hide a selected predictor

  [X] Exit the visualization tool

Selection:
```

Displaying alignments and predicted structure models

Users may wish to inspect the quality of predicted protein structures and their alignment to structural homologs. The run_visualizations.pl interactive prompt provides users with the option to view the predicted structures as-is or aligned to their structural homologs. Selecting option [M] will open the model selection prompt. An example of how to use the model selection prompt is shown below.

```
Selection: M

Which of the following predictors would you like to see viewable structural
predictions for?

          ALPHAFOLD
          RAPTORX
          ROSETTAFOLD

          Selection: RAPTORX

          Which of the following models would you like to visualize?

                ECU03_1140-m1.pdb
                ECU03_1140-m2.pdb
                ECU03_1140-m3.pdb
                ECU03_1140-m4.pdb
                ECU03_1140-m5.pdb

                Selection: ECU03_1140-m1.pdb
```

In the above example, the user selected to view model 1 for ECU03_1140 as predicted by RaptorX. The corresponding model is shown below.

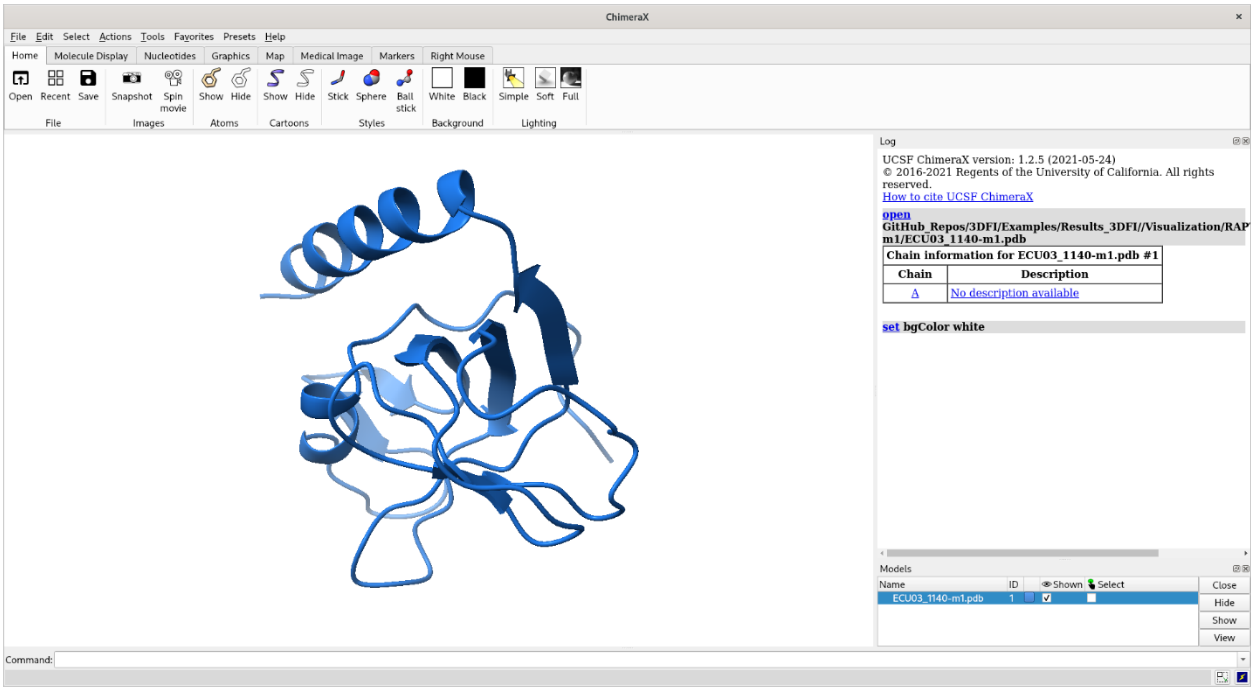

Fig. S3. Visualization of RaptorX predicted structure. Model 1 for ECU03_1140 as predicted by RaptorX is opened in ChimeraX through the use of run_visualizations.pl.

Options [1-#] are linked to the corresponding alignment files. Selecting option [1] opens the following alignment for ECU03_1140:

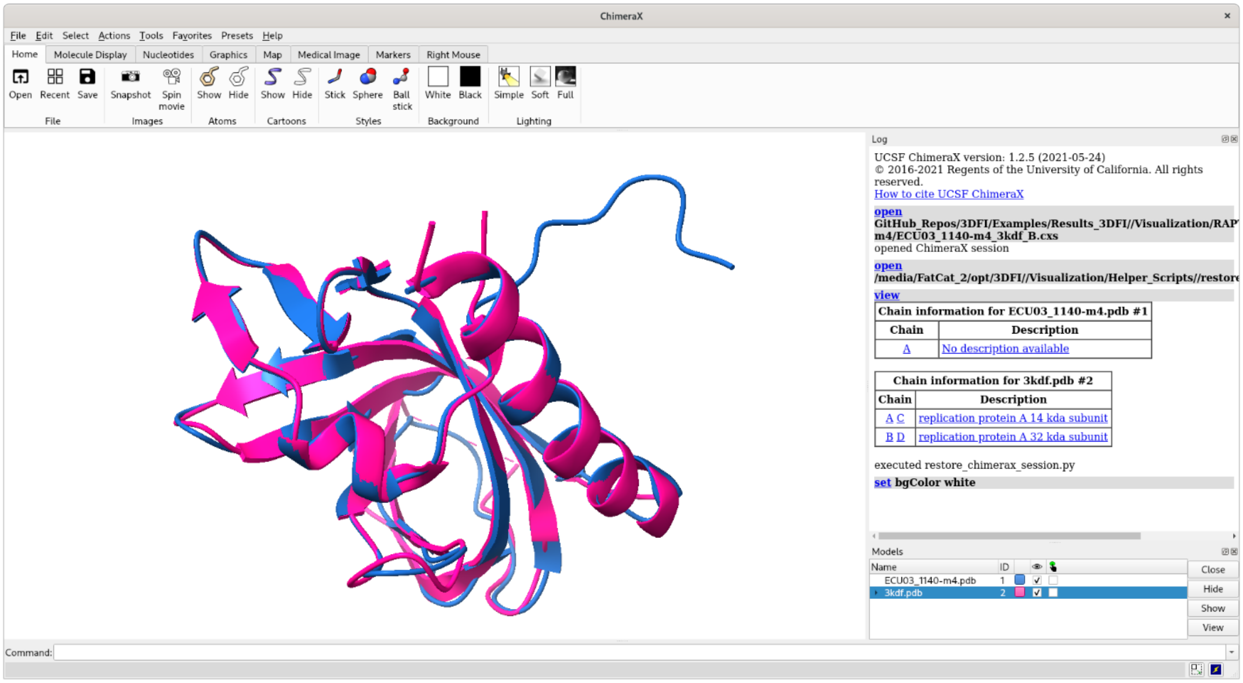

Fig. S4. Visualization of RaptorX predicted structure aligned to its structural homolog. Model 1 for ECU03_1140 as predicted by RaptorX (in blue) aligned with its structural homolog (replication protein A; in pink).

Navigating the run_visualizations.pl interactive prompt

The interactive prompt of run_visualizations.pl has various options that users may select from to customize the presentation of information. By default, run_visualizations.pl launches in 'best match mode', which displays only the top five ranked matches for a protein. To switch to 'all match mode', which displays all ranked matches, users can select option [A] in the prompt resulting in an output similar to the following:

```
### ECU03_1140 has 15 matches. ###
- Currently in all match mode
- Viewing only proteins with matches
|===============================================================================================|
  Selection   Q-Score      Predicted Structure   …   Structural Homolog Description
|===============================================================================================|
      1        0.822        RAPTORX => Model 4   …   REPLICATION PROTEIN A 32 KDA SUBUNIT
      2        0.785        RAPTORX => Model 4   …   PROTEIN (REPLICATION PROTEIN A 32 KD SUBUNIT)
-----------------------------------------------------------------------------------------------
     12        0.651       ALPHAFOLD => Model 2   …   PUTATIVE UNCHARACTERIZED PROTEIN
     13        0.632        RAPTORX => Model 2   …   CST COMPLEX SUBUNIT STN1
     14        0.535        RAPTORX => Model 5   …   PUTATIVE UNCHARACTERIZED PROTEIN
     15        0.530        RAPTORX => Model 5   …   PUTATIVE UNCHARACTERIZED PROTEIN
|===============================================================================================|
```

Users can revert back to 'best match mode' by selecting option [B].

When 3DFI is run on more than one protein, users can navigate between the results for each protein. Moving to the next or previous protein can be achieved by selecting option [N] and [P] respectively. Users can also jump to the results for a specific protein with the [J] option.

Some users may be interested in hiding results for a specific predictor. This can be achieved by selecting option [H] and following the hide prompt. The process of hiding results for RaptorX is shown below.

```
Selection: H

    Which of the following predictors you would like to hide?

            ALPHAFOLD
            RAPTORX
            ROSETTAFOLD

    Selected predictor: RAPTORX
```

Hiding the RaptorX predictor produces the following results table for ECU03_1140:

```
|=======================================================================================|
  Selection    Q-Score     Predicted Structure    …   Structural Homolog Description
|=======================================================================================|
      1       0.658     ALPHAFOLD => Model 2    …   REPLICATION PROTEIN A 32 KDA SUBUNIT
      2       0.651     ALPHAFOLD => Model 2    …   PUTATIVE UNCHARACTERIZED PROTEIN
|=======================================================================================|
```

Users can show hidden predictors by selecting the [S] option and following a similar selection prompt.

Interpreting the results of the 3DFI pipeline

The Q-score provided for identified homologs is a quantitative measurement of structural homology. While the Q-score is a good place to start looking for putative function assignment, it should not be used alone. The results of the 3DFI pipeline require careful consideration by users before functional inferences can be made.

Take for instance the highly conserved WD40 β-propeller domain shown below. This repetitive domain is present in a large number of proteins. A predicted structure containing even a partial WD40 domain will accumulate many structural homologs with relatively high Q-scores but inspecting the alignments for the predicted structure will reveal a pattern of false putative functions (false positives).

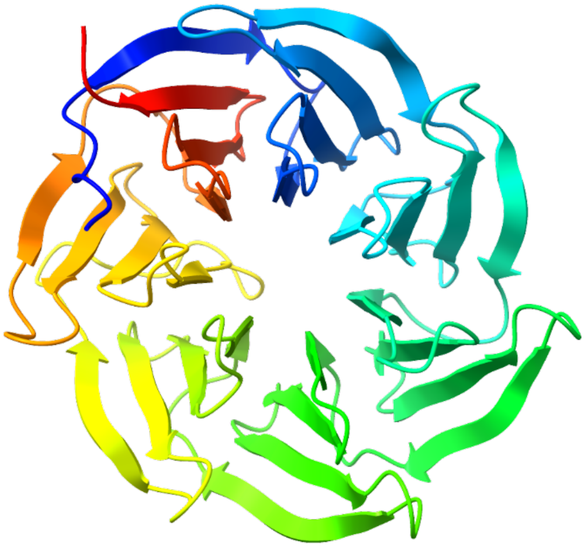

Fig. S5. The highly conserved WD40 β-propeller domain. The WD40 domain is present in a large number of proteins. Searching 'WD40' on the RCSB PDB returns 2236 structures (as of 2021-10-07) whose title contains this keyword.

Additionally, users should consider background information about the proteins present in their organism of study. Consider the structural homologs found for ECU03_1140 provided with the 3DFI pipeline:

```
|==================================================================================|
  Selection   Q-Score     Predicted Structure    …   Structural Homolog Description
|==================================================================================|
     1         0.822       RAPTORX => Model 4    …   REPLICATION PROTEIN A 32 KDA SUBUNIT
     2         0.785       RAPTORX => Model 4    …   PROTEIN (REPLICATION PROTEIN A 32 KD SUBUNIT)
     3         0.785       RAPTORX => Model 4    …   REPLICATION PROTEIN A 32 KDA SUBUNIT
     4         0.784       RAPTORX => Model 4    …   REPLICATION PROTEIN A 32 KDA SUBUNIT
------------------------------------------------------------------------------------
     9         0.670       RAPTORX => Model 1    …   Protein STN1
|==================================================================================|
```

A user could be tempted to refer to ECU03_1140 as replication protein A2 (Rpa2) based on Q-scores alone. However, if the top ranked protein alignment is inspected side by side with a slightly lower ranked alignment between ECU03_1140 and Stn1, as shown below, both Rpa2 and Stn1 appear as acceptable structural homologs and cannot be distinguished without external evidence.

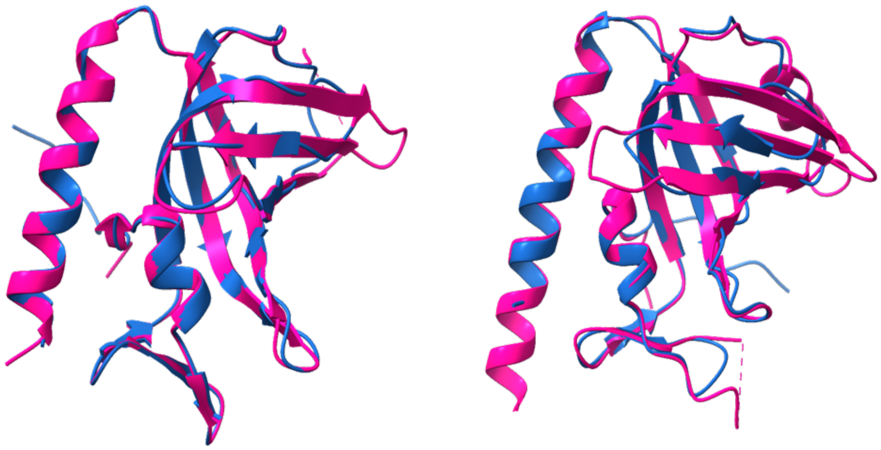

RaptorX -- Model 4
Rpa2
Q- Score: 0.822

RaptorX -- Model 1
Stn1
Q-Score: 0.670

Fig. S6. Alignment of two ECU03_1140 predicted models to their Rpa2 and Stn1 structural homologs. Model 4 and model 1 predicted by RaptorX for ECU03_1140 display similar fold quality. Additionally, the two models align to their corresponding homologs equally well.

However, a delve into the literature of Microsporidia reveals that Rpa2 has already been found in *Encephalitozoon cuniculi* using sequence-based homology approaches as another protein. By process of elimination, ECU03_1140 is likely to be Stn1 rather than Rpa2 as would be implied by the top Q-score. Stn1 is a telomere specific structural analog to Rpa2, which explains the similarity between the two structures. Thus, structural homology is a great complement to sequence-based approaches but should not be used as a standalone.
