# Supplementary material for: 3DFI: a pipeline to infer protein function using structural homology
Source: Bioinform Adv. 2021 Nov 10;1(1):vbab030. doi: 10.1093/bioadv/vbab030 (PMC9162058; doi:10.1093/bioadv/vbab030)
Supplement: vbab030_Supplementary_Data [file vbab030_supplementary_data.zip › Supplementary_Data_2_3DFI.pdf]

Supplementary Data 2: Step-by-step walkthrough of how to create custom GESAMT archives using 3DFI scripts. This walkthrough was tested on Fedora 33/34 Linux installations with 3DFI version 0.8.6. Up-to-date instructions can be found on GitHub (https://github.com/PombertLab/3DFI).

Why a custom database?

While the 3DFI pipeline utilizes the RCSB PDB as a database, GESAMT can create and query custom databases. Users interested in specific proteins (such as those in certain biological pathways) will likely desire to identify predicted structures that are relevant to their investigation rather than using an all versus all approach. To obtain a more concise and relevant list of structural homologs, users can query known proteins against a database comprised of predicted proteins.

How to create a custom database with run_GESAMT.pl

The run_GESAMT.pl script included in 3DFI can create custom databases. In the case example provided below, a custom database (a.k.a. an archive using GESAMT nomenclature) is created from the RaptorX predicted structures.

```
export GESAMT_ARCHIVE=XXX            ### Replace XXX with desired archive location
export TDFI_DB=$TDFI_HOME/Examples/Results_3DFI/Folding/RaptorX_3D

run_GESAMT.pl \
   -cpu 10 \
   -make \
   -arch $GESAMT_ARCHIVE \
   -pdb $TDFI_DB
```

How to query a custom database with run_GESAMT.pl

Proteins of interest can be queried against custom databases. PDB files obtained from RCSB often contain two or more chains and/or proteins complexed together. The latter is the case with entry 3KF6 (obtained from https://files.rcsb.org/download/3KF6.pdb), which contains both Stn1 and Ten1. The two structures can be separated into their own PDB files by using the split_PDB.pl script (3DFI/Misc) as follows:

```
split_PDB.pl \
   -p 3kf6.pdb \
   -o Split_3kf6 \
   -e pdb
```

Inspecting the files contained in Split_3kf6 reveals that 3kf6_A.pdb is Stn1, the protein that will be queried against the predicted structures in the example below. To search for Stn1 structural homologs in the RaptorX predicted structures, run_GESAMT.pl can be used.

```
export GSMT=XXX                      ## Replace XXX with desired output location

run_GESAMT.pl \
   -cpu 10 \
   -query \
   -arch $GESAMT_ARCHIVE \
   -input Split_3kf6/3kf6_A.pdb \
   -o $GSMT \
   -mode normal
```

The output file will look similar to the following:

```
#  Hit    PDB  Chain  Q-score  r.m.s.d     Seq.  Nalign  nRes    File
#  No.    code   Id                         Id.                  Name
1               A      0.6699   1.0157   0.1376    109     117   ECU03_1140-m1.pdb
2               A      0.6043   1.4564   0.1284    109     117   ECU03_1140-m4.pdb
3               A      0.5903   1.4034   0.1215    107     117   ECU03_1140-m2.pdb
4               A      0.5722   1.4517   0.1132    106     117   ECU03_1140-m3.pdb
5               A      0.4266   1.6479   0.1383     94     117   ECU03_1140-m5.pdb
6               A      0.0901   3.6652   0.0299     67     147   ECU06_1350-m3.pdb
7               A      0.0874   3.9415   0.0290     69     147   ECU06_1350-m2.pdb
8               A      0.0656   3.6478   0.0526     57     147   ECU06_1350-m5.pdb
9               A      0.0538   3.8073   0.1132     53     147   ECU06_1350-m1.pdb
10              A      0.0311   4.0631   0.1190     42     147   ECU06_1350-m4.pdb
```
